# Supplementary material for: Feed Restriction Reveals Distinct Serum Metabolome Profiles in Chickens Divergent in Feed Efficiency Traits
Source: Metabolites. 2019 Feb 25;9(2):38. doi: 10.3390/metabo9020038 (PMC6409680; doi:10.3390/metabo9020038)
Supplement: Supplementary file 1 [file metabolites-09-00038-s001.pdf]

Table S1. Dietary ingredients and chemical composition of diets (on as-fed basis).

Item	Starter<br>1 to 8 d post-hatch	Grower<br>9 to 20 d post-hatch	Finisher<br>21 to 30 d post-hatch
Ingredient (%)			
Corn	56.40	61.77	63.93
Soybean meal	37.04	31.43	29.06
Soybean oil	2.28	2.68	3.41
Monocalcium phosphate	1.46	1.41	1.24
Calcium carbonate	1.37	1.28	1.16
DL-Methionine	0.35	0.31	0.26
Natrium bicarbonate	0.20	0.21	0.14
Lysine-HCL 98	0.14	0.15	0.04
Salt	0.12	0.12	0.16
L-Threonine	0.04	0.04	0
Premix[1]	0.60	0.60	0.60
Analyzed chemical composition, g/kg dry matter			
Dry matter	882	885	879
Crude protein	228	207	194
Crude fat	51.1	47.5	61.0
Crude fiber	28.0	29.0	30.1
Crude ash	52.6	50.0	46.3
Starch	356	388	401
Sugar	55.0	49.9	45.4
Calcium	8.88	8.39	7.63
Phosphorus	6.90	7.05	6.66
ME, MJ/kg	12.0	12.0	12.4

[1]Provided per kilogram of complete starter diet (Garant - Tiernahrung GmbH, Pöchlarn, Austria): 12,500 IU of vitamin A, 5,000 IU of vitamin D_3, 75.0 mg of vitamin E, 6.0 mg of vitamin K3, 2.50 mg of vitamin B_1, 7.0 mg of vitamin B_2, 4.50 mg of vitamin B_6, 0.025 mg of vitamin B_{12}, 60.0 mg of nicotinic acid, 15.0 mg of pantothenic acid, 1.0 mg of folic acid, 0.25 mg of biotin, 1,582.065 mg of choline, 400.322 mg of choline chloride, 115.20 mg of betaine, 19.98 mg of ethoxyquin, 900.004 FTU of 6Phytase, 0.564 g of ß-glucan, 2.428 % of C 18:2, 2.804 % of polyenic acid, 30.059 mg of F-Xanto (total). Provided per kilogram of complete grower diet (Garant - Tiernahrung GmbH, Pöchlarn, Austria): 12,500 IU of vitamin A, 5,000 IU of vitamin D_3, 75.0 mg of vitamin E, 6.0 mg of vitamin K3, 2.50 mg of vitamin B_1, 7.0 mg of vitamin B_2, 4.50 mg of vitamin B_6, 0.025 mg of vitamin B_{12}, 60.0 mg of nicotinic acid, 15.0 mg of pantothenic acid, 1.0 mg of folic acid, 0.25 mg of biotin, 1,457.765 mg of choline, 400.322 mg of choline chloride, 115.20 mg of betaine, 19.98 mg of ethoxyquin, 900.004 FTU of 6Phytase, 0.618 g of ß-glucan, 2.692 % of C 18:2, 3.103% of polyenic acid, 30.866 mg of F-Xanto (total). Provided per kilogram of complete finisher diet (Garant - Tiernahrung GmbH, Pöchlarn, Austria): 5,000 IU of vitamin D_3, 75.0 mg of vitamin E, 6.0 mg of vitamin K3, 2.50 mg of vitamin B_1, 7.0 mg of vitamin B_2, 4.5 mg of vitamin B_6, 0.025 mg of vitamin B_{12}, 60.0 mg of nicotinic acid, 15.0 mg of pantothenic acid, 1.0 mg of folic acid, 0.25 mg of biotin, 1,404.717 mg of choline, 400.322 mg of choline chloride, 115.20 mg of betaine, 19.98 mg of ethoxyquin, 900.004 FTU of 6Phytase, 0.639 g of ß-glucan, 3.098 % of C 18:2, 3.572 % of polyenic acid, 31.189 mg of F-Xanto (total).

Table S2. Total feed intake (TFI), total body weight gain (TBWG), and residual feed intake (RFI) values of low and high RFI broiler chickens fed either *ad libitum* or restrictively[1].

	Ad libitum		Restrictive			*P* value		
Item	Low RFI	High RFI	Low RFI	High RFI	SEM	FL[4]	RFI	FL × RFI
TFI (g)	2337[b]	2620[a]	2110[c]	2171[c]	52.5	<0.001	0.002	0.040
TBWG (g)	1696	1684	1501	1416	41.1	<0.001	0.242	0.376
RFI (g)	-81[c]	226[a]	-67[c]	111[b]	23.0	0.033	<0.001	0.007

Data are presented as least-square means and pooled SEM. FL feed intake level. *n* = 7 per FL group, RFI rank, and sex; except for *n* = 8 high RFI *ad libitum* females. TFI, TBWG, and RFI were calculated for the experimental period from 9 to 30 days post-hatch. Different superscripts within a row indicate significant difference ($P \leq 0.05$).

[1] Siegerstetter, S.-C.; Petri, R.M.; Magowan, E.; Lawlor, P.G.; Zebeli, Q.; O'Connell, N.E.; Metzler-Zebeli, B.U. Feed restriction modulates the fecal microbiota composition, nutrient retention and feed efficiency in chickens divergent in residual feed intake. *Front Microbiol.* **2018**, *9*, 2698. doi: 10.3389/fmicb.2018.02698.

Table S3. Additional serum metabolites in low and high RFI broiler chickens fed either *ad libitum* or restrictively.

	Ad libitum feeding		Restrictive feeding			*p* Value		
Metabolite[1]	Low RFI	High RFI	Low RFI	High RFI	SEM	FL	RFI	FL × RFI
**Blood biochemistry**								
Glucose (mg/dL)	276	286	274	283	7.71	0.789	0.217	0.970
Triglycerides (mg/dL)	78.3	84.0	77.3	90.6	7.01	0.692	0.178	0.589
NEFA (mmol/L)	0.67	0.68	0.70	0.68	0.03	0.627	0.941	0.657
Alkaline Phosphatase (U/L)	669	681	701	683	34.53	0.719	0.504	0.681
**Amino acids (µmol/L)**								
Alanine	1013.7	829.2	963.8	966.5	58.42	0.458	0.126	0.115
Arginine	548.5	522.1	533.0	598.3	33.68	0.372	0.566	0.180
Aspartate	120.3	133.7	94.7	106.1	19.34	0.175	0.524	0.958
Glutamine	1038.7	920.6	979.0	977.0	52.52	0.975	0.258	0.275
Glutamate	334.2	290.9	289.0	282.8	22.42	0.237	0.270	0.408
Methionine	159.4	160.1	149.9	174.4	8.22	0.770	0.133	0.155
Phenylalanine	144.4	145.9	149.6	159.4	5.79	0.114	0.329	0.478
**Biogenic amines (µmol/L)**								
Asymmetric dimethylarginine	1.15	1.14	1.17	1.18	0.05	0.564	0.908	0.819
Alpha-aminoadipic acid	2.44	2.43	1.95	2.43	0.17	0.165	0.175	0.163
Creatinine	2.42	2.39	2.18	2.33	0.15	0.315	0.707	0.560
Histamine	0.10	0.10	0.11	0.09	0.01	0.934	0.329	0.507
Methionine sulphoxide	11.8	12.5	11.6	13.2	0.59	0.726	0.054	0.433
Putrescine	0.84	0.82	0.60	0.75	0.11	0.173	0.539	0.433
Serotonin	39.4	35.7	30.8	35.2	5.12	0.379	0.952	0.437
Spermidine	0.36	0.31	0.25	0.26	0.04	0.067	0.600	0.436
Spermine	0.29	0.27	0.29	0.24	0.03	0.613	0.279	0.751
**Acyl-carnitines (µmol/L)**								
C0	3.56	4.50	3.15	3.36	0.630	0.226	0.365	0.561
C2	0.57	0.67	0.43	0.42	0.110	0.081	0.669	0.593
C3-DC	0.060	0.081	0.055	0.065	0.013	0.424	0.237	0.702
C4:1	0.030	0.033	0.031	0.031	0.001	0.631	0.414	0.414
C14:1	0.050	0.053	0.050	0.051	0.002	0.702	0.356	0.702
C18	0.015	0.016	0.012	0.016	0.001	0.308	0.092	0.383
Sum of hexoses (mmol/L)	13.6	13.5	13.3	12.9	0.36	0.226	0.543	0.693
**Lysophosphatidylcholines (µmol/L)**								
LysoPC aC16:0	26.0	28.4	28.4	30.1	1.26	0.106	0.108	0.808
LysoPC a C18:2	13.1	14.0	13.9	15.3	0.69	0.141	0.105	0.705
**Sphingomyelins (µmol/L)**								
SM (OH) C14:1	0.50	0.47	0.52	0.53	0.024	0.119	0.654	0.274
SM (OH) C16:1	1.03	0.92	0.97	0.96	0.053	0.839	0.219	0.356
SM (OH) C22:1	2.22	2.13	2.19	2.17	0.108	0.972	0.616	0.720
SM (OH) C22:2	0.40	0.38	0.39	0.39	0.025	0.889	0.625	0.748
SM (OH) C24:1	0.56	0.57	0.55	0.64	0.030	0.257	0.111	0.165
SM C16:0	153.3	151.9	150.1	159.2	5.916	0.725	0.524	0.375

SM C16:1	0.98	0.94	0.96	1.03	0.037	0.383	0.659	0.125
SM C18:0	17.4	16.8	16.5	17.0	0.794	0.680	0.994	0.504
SM C18:1	1.28	1.36	1.24	1.31	0.059	0.464	0.230	0.902
SM C20:2	0.12	0.13	0.12	0.13	0.011	0.930	0.358	0.461
SM C24:1	16.1	17.1	15.9	17.8	0.804	0.741	0.072	0.558
SM C26:0	0.013	0.008	0.013	0.017	0.003	0.196	0.960	0.204
SM C26:1	0.017	0.027	0.022	0.027	0.007	0.787	0.293	0.698
**Glycerophosphatidylcholines (μmol/L)**								
PC aa C28:1	0.33	0.27	0.28	0.28	0.017	0.203	0.170	0.068
PC aa C30:0	2.3	2.3	2.3	2.4	0.088	0.678	0.800	0.249
PC aa C32:2	2.4	2.3	2.4	2.7	0.120	0.107	0.792	0.076
PC aa C34:2	291.8	284.9	300.4	306.8	10.521	0.153	0.983	0.528
PC aa C34:3	15.1	14.5	15.6	16.1	0.730	0.164	0.982	0.425
PC aa C34:4	1.2	1.1	1.0	1.0	0.053	0.148	0.577	0.241
PC aa C36:2	344.3	357.5	361.9	385.1	13.119	0.092	0.172	0.703
PC aa C36:4	170.9	163.7	158.8	159.0	6.138	0.178	0.569	0.547
PC aa C36:6	0.42	0.44	0.38	0.44	0.024	0.384	0.144	0.354
PC aa C38:0	1.04	0.93	1.07	0.94	0.090	0.850	0.174	0.883
PC aa C38:1	0.97	1.01	1.01	1.06	0.070	0.504	0.564	0.957
PC aa C38:6	42.6	44.1	39.4	43.6	2.222	0.400	0.201	0.553
PC aa C40:2	0.87	0.78	0.91	0.81	0.066	0.653	0.154	0.964
PC aa C40:3	1.53	1.47	1.46	1.40	0.076	0.318	0.429	0.997
PC aa C40:4	11.6	11.4	11.3	10.6	0.558	0.369	0.398	0.687
PC aa C40:6	24.4	26.3	24.0	26.4	1.545	0.927	0.177	0.857
PC aa C42:0	0.081	0.069	0.071	0.055	0.009	0.203	0.139	0.846
PC aa C42:1	0.11	0.10	0.10	0.10	0.006	0.582	0.632	0.441
PC aa C42:2	0.25	0.24	0.26	0.27	0.011	0.244	0.993	0.276
PC ae C34:0	0.98	0.95	0.93	0.95	0.043	0.509	0.978	0.551
PC ae C34:1	7.28	7.28	6.69	6.93	0.381	0.226	0.749	0.747
PC ae C34:2	8.66	8.20	8.16	7.99	0.462	0.442	0.495	0.747
PC ae C34:3	1.42	1.34	1.35	1.34	0.066	0.575	0.479	0.548
PC ae C36:0	0.40	0.39	0.38	0.38	0.018	0.331	0.901	0.822
PC ae C36:1	2.42	2.50	2.62	2.54	0.170	0.472	0.985	0.643
PC ae C36:2	5.88	5.69	5.76	5.62	0.248	0.702	0.513	0.927
PC ae C36:3	3.65	3.59	3.29	3.27	0.177	0.059	0.835	0.888
PC ae C36:5	2.44	2.28	2.29	2.16	0.162	0.386	0.370	0.929
PC ae C38:1	1.13	1.19	1.31	1.10	0.147	0.727	0.607	0.354
PC ae C38:2	2.62	2.62	2.73	2.64	0.152	0.687	0.748	0.770
PC ae C38:3	1.96	2.07	1.89	1.78	0.152	0.242	1.000	0.483
PC ae C38:4	12.6	11.6	10.9	10.3	0.843	0.088	0.355	0.869
PC ae C38:5	9.66	8.69	8.26	7.75	0.639	0.074	0.250	0.725
PC ae C38:6	2.32	2.06	2.09	1.98	0.163	0.351	0.261	0.672
PC ae C40:2	0.49	0.50	0.52	0.49	0.034	0.928	0.774	0.572
PC ae C40:3	0.77	0.85	0.76	0.56	0.156	0.338	0.721	0.378

PC ae C40:5	2.94	2.62	2.55	2.31	0.179	0.057	0.120	0.836
PC ae C40:6	1.83	1.62	1.60	1.51	0.111	0.123	0.172	0.579
PC ae C42:0	0.82	0.79	0.77	0.77	0.029	0.271	0.581	0.752
PC ae C42:1	0.89	0.84	0.81	0.75	0.046	0.072	0.245	0.968
PC ae C42:2	0.60	0.59	0.54	0.56	0.029	0.107	0.814	0.642
PC ae C44:3	0.09	0.09	0.08	0.09	0.006	0.097	0.792	0.801
PC ae C44:6	0.06	0.05	0.06	0.06	0.004	0.658	0.516	0.496
**White blood cells (%)**								
Monocytes	1.22	1.65	0.95	1.12	0.262	0.135	0.260	0.630
Heterophils	14.1	14.8	11.8	10.5	1.689	0.055	0.848	0.553
Basophils	0.16	0.08	0.11	0.07	0.055	0.574	0.329	0.736
Heterophil-to-lymphocyte ratio (%)	17.2	20.9	14.0	12.0	0.036	0.102	0.820	0.430

Data are presented as least-square means and pooled SEM. RFI, residual feed intake; FL feed intake level. *n* = 7 per FL group, RFI rank, and sex; except for *n* = 8 high RFI *ad libitum* females. RFI was calculated for the experimental period from 9 to 30 days post-hatch.

[1] C0, free carnitine; C2, acetylcarnitine; C3-DC, malonylcarnitine; C4:1, butenyl-carnitine; C14:1, tetradecenoyl-carnitine; C18, octadecanoyl-carnitine; LysoPC a C, with acyl residue sum C; NEFA, non-esterified fatty acids; PC aa C, phosphatidylcholine with diacyl residue sum C; PC ae C, phosphatidylcholine with acyl-alkyl residue sum C; SM (OH) C, hydroxysphingomyelin with acyl residue sum C; SM C, sphingomyelin with acyl residue sum C.
